# Anæsthetics

**Published:** 1883-01

**Authors:** 


### Anæsthetics.

A correspondent of the *Lancet and Clinic* writing from Vienna, says:

"The anæsthetic used here and in all other parts of the vast hospital is not ether, but a mixture of alcohol and ether, one part each, and three parts of chloroform." For this mixture it is claimed that it is more prompt than ether alone, is more agreeable as to odor, and does not produce the nausea so often experienced, and it is attended with far less danger than chloroform alone; indeed, is not more so than pure ether. The anæsthetic is administered in small quantities at a time on a piece of white flannel spread on a wire frame sufficiently large to cover the nose and mouth.

A handle is attached by which it can be conveniently held.

